# Smyd1 Orchestrates Early Heart Development Through Positive and Negative Gene Regulation

**DOI:** 10.3389/fcell.2021.654682

**Published:** 2021-04-01

**Authors:** Zhen Wang, Robert J. Schwartz, Jing Liu, Fei Sun, Qi Li, Yanlin Ma

**Affiliations:** ^1^Hainan Provincial Key Laboratory for Human Reproductive Medicine and Genetic Research, The First Affiliated Hospital of Hainan Medical University, Hainan Medical University, Haikou, China; ^2^Key Laboratory of Tropical Translational Medicine of Ministry of Education, Hainan Medical University, Haikou, China; ^3^Hainan Provincial Clinical Research Center for Thalassemia, The First Affiliated Hospital of Hainan Medical University, Hainan Medical University, Haikou, China; ^4^Haikou Key Laboratory for Preservation of Human Genetic Resource, The First Affiliated Hospital of Hainan Medical University, Hainan Medical University, Haikou, China; ^5^Department of Biology and Biochemistry, University of Houston, Houston, TX, United States; ^6^Department of Reproductive Medicine Center, Zhengzhou University, Zhengzhou, China

**Keywords:** Smyd1, heart, ISL-1, ANF, ASH2L

## Abstract

SET and MYND domain-containing protein 1 (Smyd1) is a striated muscle-specific histone methyltransferase. Our previous work demonstrated that deletion of Smyd1 in either cardiomyocytes or the outflow tract (OFT) resulted in embryonic lethality at E9.5, with cardiac structural defects such as truncation of the OFT and right ventricle and impaired expansion and proliferation of the second heart field (SHF). The cardiac phenotype was accompanied by the downregulation of ISL LIM Homeobox 1 (Isl1) and upregulation of atrial natriuretic factor (ANF). However, the mechanisms of Smyd1 regulating Isl1 and ANF during embryonic heart development remain to be elucidated. Here, we employed various biochemical and molecular biological approaches including chromatin immunoprecipitation polymerase chain reaction (ChIP-PCR), pGL3 fluorescence reporter system, and co-immunoprecipitation (CoIP) and found that Smyd1 interacted with absent small homeotic-2-like protein (ASH2L) and activated the promoter of Isl1 by trimethylating H3K4. We also found that Smyd1 associated with HDAC to repress ANF expression using trichostatin A (TSA), a deacetylase inhibitor. In conclusion, Smyd1 participates in early heart development by upregulating the expression of Isl1 and downregulating the expression of ANF.

## Introduction

Congenital heart disease affects 0.8–1 child per 100 live births and is responsible for the majority of prenatal deaths (Bonnet, 2017). Although contribution has been made in drawing the molecular mechanisms and genetic basis of heart formation and malformation, the fundamental causes of congenital heart disease are still unclear. Previous studies have shown that serum response factor (SRF), NK2 homeobox 5 (Nkx2.5), GATA binding protein 4 (GATA4), and T-box transcription factor 20 (Tbx20) are important for both first and second heart fields, while Hand2, Isl1, Myocyte Enhancer Factor 2C (Mef2c), and fibroblast growth factor 10 (Fgf10) are important for the formation of the second heart field (Rochais et al., 2014; Quaranta et al., 2018; Chen et al., 2019; Han et al., 2019). Hand1 and Tbx5 are more important for generation of the first heart field. In particular, Isl1 marks cardiac progenitors with vascular endothelial growth factor receptor 2 (VEGFR2, Flk-1) and Nkx2.5, which could be isolated, propagated, and differentiated into endothelial, smooth muscle, and cardiomyocyte (Cai et al., 2003; Kattman et al., 2006; Moretti et al., 2006). Those findings suggest that Isl1 is critical for cardiac progenitor commitment and early heart development.

Histone modifications are the main components of epigenetics and play important roles in regulating mammalian development (Skvortsova et al., 2018). These modifications are involved in chromatin remodeling and gene activation or silencing. For example, trimethylation of histone 3 lysine 4 (H3K4me3) is highly correlated with transcriptional initiation and plays a key role in regulating developmental potential during differentiation of embryonic stem (ES) cells and multiple progenitor cells (Berger, 2002). However, the precise mechanisms of how those histone modifications correlate with individual gene expression are not well understood.

Smyd1 is a striated muscle-specific histone methyltransferase, regulates cardiac energy metabolism (Warren et al., 2018), and is critical for heart generation (Gottlieb et al., 2002; Warren et al., 2018; Chow et al., 2019). A recent study revealed that Smyd1 positively regulates metabolism by targeting Perm1 (Oka et al., 2020). However, the molecular mechanism of Smyd1 in heart development is still not clear. Although yeast two-hybrid assay revealed that Smyd1 might repress gene expression by interacting with HDACs (Gottlieb et al., 2002), there was no evidence showing its downstream targets. On the other hand, Smyd1 has been shown to catalyze H3K4me3 through its split SET domain *in vitro* (Tan et al., 2006), but the significance of H3K4me3 induced by Smyd1 *in vivo* has yet to be determined.

In the present study, we found that Smyd1 bound to the *Isl1* promoter and promoted H3K4me3 on that region, resulting in the activation of Isl1. In addition, ASH2L (Steward et al., 2006), an important non-catalytic cofactor for trimethylation, associates with Smyd1 to activate the *Isl1* promoter. Smyd1 also bound to the *ANF* promoter and inhibited the expression of *ANF* through association with HDACs. These findings identified Smyd1 as a critical epigenetic factor in early heart morphogenesis through both positive and negative regulation of gene expression.

## Materials and Methods

### Mice

Floxed–*Smyd1* mice were provided by Dr. Philip Tucker. Two loxp sites were targeted to introns 1 and 3 of *Smyd1*. *Nkx2.5-Cre* mice were created in our laboratory by a Cre knock-in into the endogenous *Nkx2.5* locus, replacing the endogenous *Nkx2.5* (Moses et al., 2001). Homozygous floxed–*Smyd1* mice were crossed with *Nkx2.5-Cre* mice to generate *Nkx2.5*^Cre+^; *Smyd1*^*flox*/+^ mice, which were then crossed with *Smyd1*^flox/flox^ mice to produce homozygous *Nkx2.5^Cre+^*; *Smyd1^flox/flox^* embryos (Cko embryos). All experiments were performed in compliance with the guidelines of the Animal Care and Use Committee of Hainan Medical University.

### Whole-Mount RNA *in situ* Hybridization

Whole-mount RNA *in situ* hybridization was carried out as previously described (Ai et al., 2007). Probes for *Smyd1* were generated as we previously described (Niu et al., 2008). Detailed probe information is available upon request. All experiments were repeated at least three times to ensure statistically relevant findings.

### Lacz Staining of Mouse Embryos

Briefly, E9.5 mouse embryos were fixed in formaldehyde for 15 min at room temperature, rinsed in detergent for 15 min at room temperature three times, and then subjected to β-gal staining for overnight at dark. After staining, embryos were washed and fixed with 4% paraformaldehyde for 1 h at room temperature and then washed and stored at 4°C.

### Immunofluorescence (IF)

Mouse hearts were fixed in 4% paraformaldehyde for 4–6 h, dehydrated in ethanol, and embedded in paraffin, which were sectioned (5–7 μm thickness) on a microtome. Sections were incubated with bovine serum albumin for 20 min at 37°C and then incubated with either rabbit anti-human ASH2L polyclonal antibody (Abclonal) or mouse anti-human Smyd1 (Santa Cruz Biotech) at 4°C for overnight. Samples were then washed in PBS three times for 15 min at room temperature and incubated with the secondary antibody Alexa Fluor 488 goat anti-mouse IgG (Abcam) and Alexa Fluor 568 goat anti-rabbit IgG (Abcam). Sections were stained with (4,6-diamidino-2-phenylindole) (DAPI) for 10 min and mounted on slides with 50% glycerol in PBS. The IF images were captured by a confocal microscope (Olympus, FV1000, Japan).

### Chromatin Immunoprecipitation (ChIP)

Chromatin immunoprecipitation assays were carried out using Upstate EZ ChIP kit (Upstate Biotechnology) according to the protocol provided by the manufacturer’s protocol with slight modifications. Briefly, heart tissues from 45 E9.5 control and *Smyd1* Cko embryos were homogenized for H3K4me3 and H3K27me3 ChIP analysis. Antibodies against Smyd1 were provided by Dr. Philip Tucker. Anti-H3K4me3 and anti-H3K4me3 antibodies were purchased from Abcam. RNA polymerase II was provided by Upstate EZ ChIP kit. IgG (Santa Cruz) was used as a negative control in ChIP. The sequences of primers used for ChIP assays were shown in Tables 1, 2. Real-time PCR was carried out using Brilliant qPCR reagent at the Mx3000P^TM^ PCR Systems (Stratagene). For ChIP experiments, 1 μl diluted (1:5) ChIP DNA or input DNA was used as a template, and relative fold enrichments were determined by the 2^ΔCT^ method described in the Applied Biosystems User Bulletin. Ratios were determined from three independent qPCR.

**TABLE 1:** Isl1 ChIP-qPCR primers.

Name	Sequence
Isl1P1 5′primer	5′-CACTTTCATCACCCGATCCT-3′
Isl1P1 3′ primer	5′-TGTGAGCAGGTCTTCCACAG-3′
Isl1P2 5′primer	5′-TCATGGTCAACCTGGCTGTA-3′
Isl1P2 3′ primer	5′-CGTGAGCAGGATGGAAAGTT-3′
Isl1P3 5′primer	5′-AGATGCAGCCAACATCTCCT-3′
Isl1P3 3′ primer	5′-TTTTAGTATGAAGGCGTGAGCA-3′
Isl1P4 5′primer	5′-GAGGGGGTGGTGGACAAG-3′
Isl1P4 3′ primer	5′-GGCGCAGCTGTTCTGATTAT-3′
Isl1TSS 5′primer	5′-ATGGAGCTGAGTTGGAGCTG-3′
Isl1TSS 3′primer	5′-TCTGGGAGTCCGATTTAAGC-3′
Isl13′UTR 5′primer	5′-CGACCCAGTCAATGGAAACT-3′
Isl13′UTR 3′primer	5′-TGGGCTTAGGGTTTTGTTTG-3′
Isl1inter 5′primer	5′-ATAGACATCCCAGCGTTGCT-3′
Isl1inter 3′primer	5′-CAATCAAACCCGCTCATTTT-3′

**TABLE 2:** ANF ChIP-qPCR primers.

Name	Sequence
ANFP15′primer	5′-CCTGACTGCTAACAGGGACA-3′
ANFP13′primer	5′-TGTCAGGGGCTCCAAATAAG-3′
ANFP25′primer	5′-ATCGCTTTATCGCTGCAAGT-3′
ANFP23′primer	5′-TCAGCTTTTGTCCGTCACTG-3′
ANFP35′primer	5′-CCTAAGCCCTTGTGGTGTGT-3′
ANFP33′primer	5′-CAGAGTGGGAGAGGCAAGAC-3′

### Promoter Subcloning and Expression Vectors

To generate the luciferase reporter of the mouse *Isl1* promoter, a 1.4-kb genomic DNA fragment (−1203 to +204) of the mouse *Isl1* 5′-promoter was subcloned into the KpnI and XhoI sites of the pLG3-basic vector (Promega). The primers were

5′ primer 5′-CCCGGGGGTACCGGCAAAAGTTACCGGT GAGA-3′ and

3′ primer 5′-CCCGGGCTCGAGAGAGAGGATGCTGGTGC TGT-3′.

Point mutations of the Smyd1-binding site in the ANF 5′-promoter region were purchased from IGE Biotechnology, and mutations on the *Isl1* 5′-promoter were generated using the QuikChange sited-directed mutagenesis kit (Stratagene) according to the manufacturer’s protocol. The primers for point mutations at site SB3 were:

5′ primer 5′-CTCTTCTTTTGCAACAGACAGACCCTGCTC ACGCCTTCATAC-3′

3′ prime 5′-GTATGAAGGCGTGAGCAGGGTCTGTCTGTT GCAAAAGAAGAG-3′

The primers for point mutations at site SB4 were:

5′ primer 5′-GAAAGCTAACCTCAGGGGAAAAAAGTAG GCGCCCCACTCTTC-3′

3′ primer 5′-GAAGAGTGGGGCGCCTACTTTTTTCCCCT GAGGTTAGCTTTC-3′

Mouse *ASH2L* cDNA were obtained from ATCC and subcloned into the pCDNA3.1 expression vector. The *ANF* luciferase reporter construct and *HDAC* expression vectors were generated as previously described (Wang et al., 2005). *PCDNA3.1-Myc-Smyd1* expression vectors and fragment mutants were provided by Dr. Deepak Srivastava. *Smyd1* HMTase-inactive mutant and PBS-Y234F were provided by Dr. Philip Tucker. Y234F was subcloned into the PCDNA3.1 vector at ClaI and XbaI sites. The primers for PCDNA3.1-Y234F cloning were:

5′ primer 5′-CCCGGGATCGATATGGAGAACGTGGAGG TCTT-3′

3′ primer 5′-CCCGGGTCTAGAGGGTCACTGCTTCTT ATGGAACAG-3′

### Cell Culture and Transient Transfections

C2C12 and CV1 cells were maintained in Dulbecco’s modified Eagle’s medium supplemented with 10% fetal bovine serum (Hyclone). Cotransfections were carried out using Lipofectamine^TM^ 2000 (Invitrogen) in OPTImum medium (Gibco) 24 h after plating. At 16 h post-transfection, the medium was changed to the regular medium. Cells transfected with plasmids or the control vectors exhibited green fluorescence under fluorescence microscopy, and the transfection efficiency was about 80%. In HDAC inhibition experiments, 100 or 150 ng/ml trichostatin A (TSA) was added 24 h after transfection. Cells were harvested 40 h after transfection, and luciferase activity assays were performed with BD *Monolight 3010*. The data shown for transfections are the means from at least three independent experiments.

### Coimmunoprecipitations Assay (Co-IP)

Co-IP assays were performed as previously described (Sepulveda et al., 2002). Co-IP of Myc-Smyd1 WT or its mutants and V5-ASH2L were carried out using the whole-cell lysate of CV1 cells overexpressing the relevant proteins. The coimmunoprecipitated products were analyzed by Western blot with anti-MYC or anti-V5 antibodies (Santa Cruz Biotech).

### Gel Filtration

V5-tagged ASH2L and MYC-tagged Smyd1 were cotransfected to HeLa cells. 40 h after transfection, whole-cell lysates were collected and stored at −80°C till used. The complex formation was determined by gel filtration performed on the FPLC equipped with a Superdex 200 10/300GL column. Briefly, the column was equilibrated with buffer A (20 mM Tris pH 8.0, 150 mM NaCl, and 1 mM DTT) and calibrated by standard molecular weight markers (Sigma MW-GF-200). The calibration curve was obtained with the method provided by Sigma Technical Bulletin (MW-GF-200). Cell lysates (100 μl, 5 mg/ml) were loaded into the column and run on buffer A at 0.75 ml/min. The fraction collector was set at 1 ml/fraction and turned on after 7.0 ml was eluted out. After the 10th fraction, the fraction collector was switched off. Each fraction was precipitated with 10% TCA and run in SDS-PAGE and analyzed by Western-blot.

### Nuclear Extracts and Electrophoretic Mobility Shift Assays (EMSAs)

Nuclear proteins were extracted from CV1 cells which overexpressed MYC-tagged Smyd1 protein. Nuclear extract preparation and EMSA were carried out following the procedure described previously (Charron et al., 1999). Briefly, the double-stranded oligonucleotide containing the WT Smyd1-binding motif of the mouse *Isl1* promoter was labeled with [α-^32^P] dCTP. 1 μg of extracted proteins was incubated with 50,000 cpm of the ^32^P-labeled *Isl1* probe at room temperature for 20 min in a buffer containing 1 μg poly dI-dC, 120 mM KCl, 25 mM MgCl_2_, 20 mM Tris–HCl [pH 7.9], 2 mM DTT, and 2 mM EDTA. In cold probe competition experiments, cold WT or MUT probes were added to the binding mixture at different ratios (from 5× to 200×). After a 15-min incubation on ice, labeled probes were added, and the samples were further incubated on ice for 20 min. In antibody competition experiments, 2 μg anti-MYC antibody was added to the reaction tubes for an additional 20-min incubation. A nondenaturing 5% polyacrylamide gel was used to separate the protein–DNA complexes. After electrophoresis 2.0 h at RT, the gels were dried at 80°C for 45 min and exposed to X-ray films.

### Statistical Evaluation

Data are presented as mean ± SD. Student’s *t* test was used for statistical comparisons when appropriate. A *p* value < 0.05 was considered statistically significant.

## Results

### Expression of *Smyd1* During Early Heart Development

**FIGURE 1:**
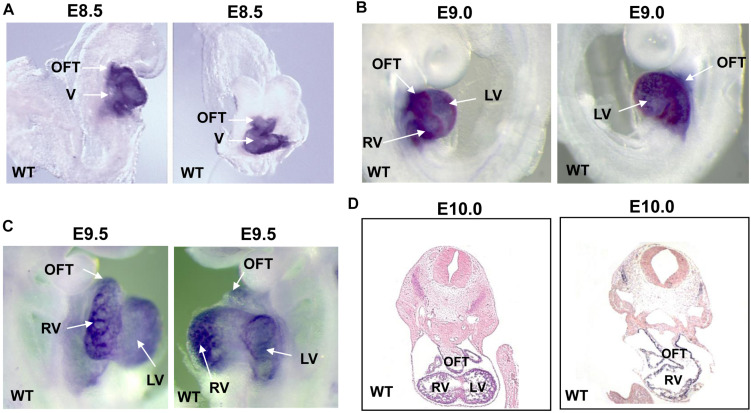
The expression of *Smyd1* in mouse embryos. **(A–C)** Whole-mount *in situ* hybridization reveals the specific expression of *Smyd1* in hearts at E8.5, E9.0, and E9.5. **(D)** *In situ* analysis reveals the expression of mouse *Smyd1* in transverse sections at E10.0. HT, heart tube; OFT, outflow tract; LV, left ventricle; V, ventricle; RV, right ventricle.

In order to investigate the temporal and spatial expression of Smyd1 during embryogenesis, we performed whole-mount *in situ* hybridization on embryos collected between E8.5 and E10.0 using a Smyd1 probe. At E8.5–9.5, high Smyd1 expression was observed in the outflow tract, ventricle, and atria (Figures 1A–C; Rasmussen et al., 2015). We also performed staining on the transverse sections at E10.0 and found that *Smyd1* was highly expressed in the myocardium (Figure 1D).

### Smyd1-Activated Isl1 Promoter

**FIGURE 2:**
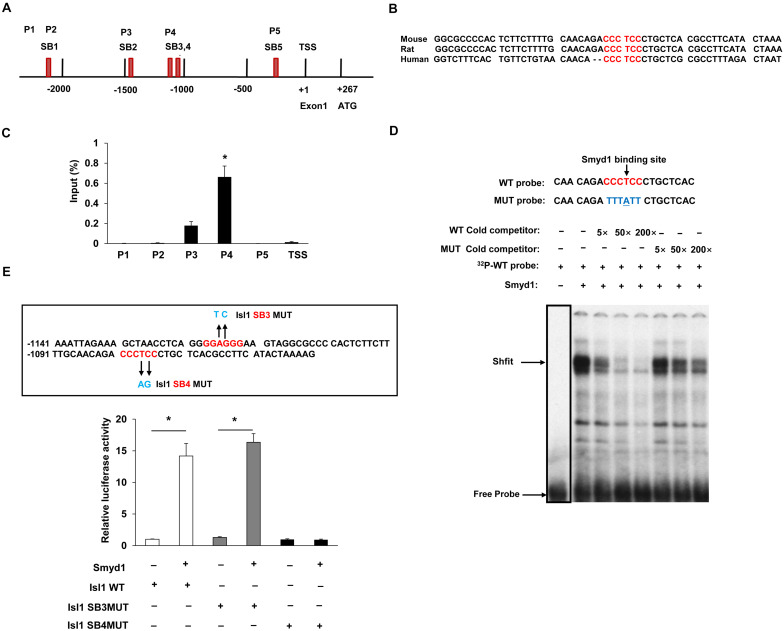
Smyd1 binds to the promoter of *Isl1*. **(A)** The locations of the primer sets (P1-TSS) used for Smyd1 ChIP-qPCR are indicated. **(B)** The sequence of the mouse *Isl1* gene containing the SB4-binding site is shown and highly conserved across mouse, rat, and human. **(C)** ChIP-qPCR revealed that Smyd1 bound to the P4 region avidly. **(D)** EMSA was performed using nuclear extracts from CV1 cells overexpressing Smyd1. The radiolabeled SB4 site shown was used as a probe. WT and MUT competitors were used at amounts from 5 × to 200 × fold molars. **(E)** Sequences that were mutated on the Isl1 promoter were indicated by arrows. Smyd1 expression vectors were cotransfected with pLG3-Isl1-WT or mutant reporter (Isl1 SB4MUT or Isl1SB3MUT) into C2C12 cells. Data are shown as mean ± SD for three independent experiments. *p < 0.01 (Student’s *t* test).

Earlier studies have suggested that knockout of Smyd1 reduced the expression of several factors required for secondary heart field formation, such as Isl1 and ANF (Rasmussen et al., 2015); however, the mechanisms behind the regulation of the expression of Isl1 and ANF by Smyd1 during embryogenesis remain to be fully elucidated. To determine whether *Isl1* was directly targeted by Smyd1, we performed ChIP–qPCR analysis with cardiac tissues from *Smyd1* Cko and control embryos. Previous studies showed that Smyd3, a member of the Smyd family, could bind to the DNA of promoter regions through the MYND-zinc-finger domain. Since Smyd1 also has a conserved MYND-zinc-finger domain and is highly homologous with Smyd3 (Du et al., 2014), it strongly suggests that Smyd1 has similar DNA-binding ability. To test this possibility, we analyzed the 3-kb promoter sequences of *Isl1* and identified 2 sites of CCCTCC and 3 sites of GGAGGG, which were potential binding sites for Smyd1. These sites were named as SB1 to SB5, respectively. All of the primers and their genetic locations are shown in Figure 2A. Among them, the core sequences of SB4 are 100% conserved in mouse, rat, and human (Figure 2B). However, the other sites are not 100% conserved. This highly suggests that the SB4 site is functionally important. To determine whether this site was a binding site for Smyd1, we designed 5 primers for ChIP-qPCR analysis according to those sites.

Our results demonstrated that Smyd1 was enriched around the 1.1-kb upstream of the TSS (P4). That fragment contained two candidate sequences, SB3 and SB4 (a “GGAGGG” site at −1119 to −1114 bp and a “CCCTCC” site at −1081 to −1075 bp). We also observed that the Smyd1 binding to other regions was much weaker than to P4 (Figure 2C).

To further determine whether the SB4 site is a binding site for Smyd1, we performed EMSA using double-stranded oligonucleotide probes. Each probe corresponded to a single potential binding sequence. With the nuclear extracts purified from CV1 cells transfected with a *Myc-Smyd1* expression plasmid, the probe which contained the SB4 site bound to Smyd1 avidly. This DNA–protein complex was reduced by an anti-MYC antibody and was abolished in the presence of an excess of the cold cognate DNA sequence as a competitor, while a mutant probe failed to compete for Smyd1 binding (Figure 2D). These results suggested that Smyd1 bound to SB4. In contrast, very weak or no binding was observed with the probe harboring the SB3 site. Hence, the SB4 “CCCTCC” site (−1081 to −1075 bp) is the critical site for Smyd1 binding on the *Isl1* promoter.

We next examined whether Smyd1 upregulates Isl1 expression at the transcriptional level by performing mouse Isl1 Luc promoter reporter activation assays. A region from −1203 to +208 bp relative to the TSS of the mouse Isl1 promoter was subcloned to the pLG3 luciferase reporter. We cotransfected the Smyd1 expression vector together with the pGL3-Isl1-WT reporter into C2C12 cells and found that the pGL3-Isl1-WT reporter was activated by Smyd1 in a dose-dependent manner. As expected, the mutation that disrupted Smyd1 binding to SB4 also reduced the activation of the Isl1-WT reporter by Smyd1, while the mutation on the SB3 site did not affect the activation of pLG3-Isl1 by Smyd1 (Figure 2E). These findings suggest that SB4 but not SB3 is important for Smyd1-dependent transcriptional activity of the Isl1 gene, which was consistent with the findings obtained from ChIP-qPCR and EMSA.

Taken together, these findings show that Smyd1 directly regulates *Isl1* expression through binding to SB4 on the *Isl1* promoter.

### Smyd1 Was Responsible for H3K4me3 in the P4 Region of the *Isl1* Promoter

**FIGURE 3:**
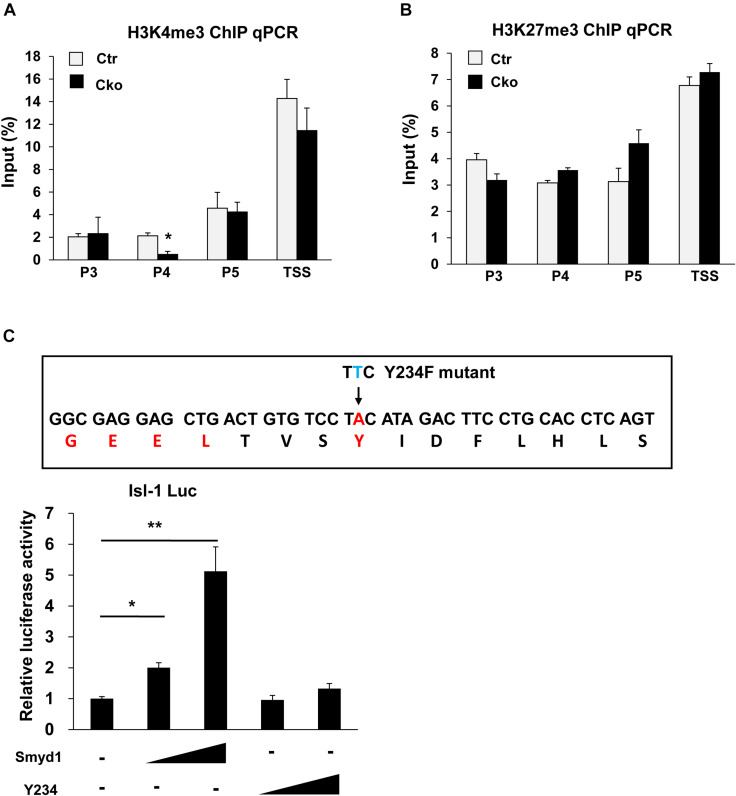
Smyd1 was responsible for H3K4me3 in the P4 region of the Isl1 promoter. **(A,B)** ChIP-qPCR for the H3K4me3 and H3K27me3 level on the different regions of the Isl1 promoter. **(C)** Sequences that were mutated on mouse Smyd1 cDNA to generate a mutation in the HMT catalytic domain of Smyd1. Amino acid Y234 was mutated to F (Y234F). Smyd1 WT or Y234F expression vectors were cotransfected with the pLG3-Isl1 reporter. Data are shown as mean ± SD for three independent experiments. *p < 0.01, **p < 0.01 (Student’s *t* test).

H3K4me3 is critical for gene activation, while H3K27me3 is related to gene repression. H3K4me3 and H3K27me3 have been found tied to poise genes as “bivalent domains” to regulate gene expression in development, especially during stem cell differentiation. Smyd1 contains a split SET domain, which has potential to trimethylate H3K4 (Tan et al., 2006). Therefore, we performed ChIP analysis using anti-H3K4me3 and anti-H3K27me3 antibodies to investigate both the H3K4me3 and H3K27me3 status on the *Isl1* promoter in control and Smyd1 Cko mice. Knockout of Smyd1 significantly reduced the H3K4me3 level in the P4 region but not that in other regions (Figure 3A), indicating that Smyd1 was responsible for the H3K4me3 in the P4 region. Surprisingly, the H3K4me3 level in the TSS region was not affected, suggesting that other H3K4 methytransferases were responsible for H3K4me3 on the TSS region of the *Isl1* promoter. We also found that knockout of Smyd1 did not alter the H3K27me3 levels on the regions between P3 and TSS of the Isl1 promoter compared with the control (Figure 3B), suggesting that Smyd1 was not responsible for H3K27me3 on the *Isl1* promoter. Meanwhile, to determine whether the HMTase activity of Smyd1 is involved in the regulation of *Isl1*, HMTase-inactive mutant *Smyd1* (*Y234F*) was transfected with the *pLG3-Isl1-WT* reporter as well. The mutant Smyd1 failed to activate the *Isl1* promoter (Figure 3C). Hence, HMTase activity of Smyd1 is required for Smyd1 to activate the Isl1 promoter.

### ASH2L Associated With Smyd1 to Activate the *Isl1* Promoter

Set methyltransferases usually work together in multi-proteins (Bergamin and Couture, 2016; Sha et al., 2020). ASH2L is one of the important non-catalytic cofactors of set-containing proteins and required for the trimethylation of H3K4. Furthermore, ASH2L is involved in muscle progenitor cell differentiation through association with diverse myogenic transcriptional factors, such as *Mef2d* and *Pax7* (Rampalli et al., 2007; McKinnell et al., 2008; Tsai et al., 2019). We asked if there was any relationship between ASH2L and Smyd1.

**FIGURE 4:**
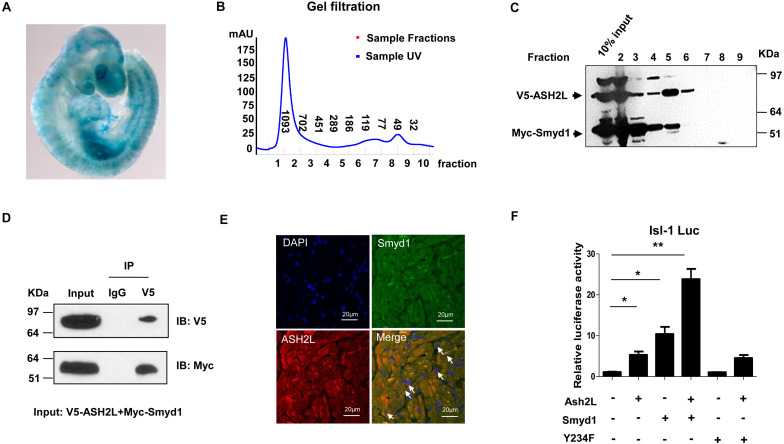
ASH2L interacts with Smyd1. **(A)** Lacz staining shows the expression pattern of ASH2L at E9.5. **(B)** Superdex 200 gel filtration revealed formation of a complex containing ASH2L and Smyd1. **(C)** Western blot detected ASH2L and Smyd1. **(D)** V5-tagged ASH2L and Myc-tagged Smyd1 were immunoprecipitated with an anti-V5 antibody. Normal IgG was used as a control. **(E)** Smyd1 and ASH2L locations in murine heart sections were determined by immunofluorescence. They were co-expressed in the nuclei and cytoplasm of cardiomyocytes with local overlapping regions (arrow). **(F)** Smyd1 WT or Y234F was cotransfected with ASH2L and pLG3-Isl1-WT luciferase reporter into C2C12 cells. The scale is 10 μm. Data are shown as mean ± SD for three independent experiments. *p < 0.05, **p < 0.01 (Student’s *t* test).

To track the expression pattern of ASH2L, we performed whole-mount Lacz staining using ASH2L-β-gal trap in embryos and observed that ASH2L was expressed in heart and somites as well as other tissues (Figure 4A). *Myc*-tagged *Smyd1* and *V5* tagged *ASH2L* were cotransfected into CV1 cells. The physical association between Smyd1 and ASH2L was determined by gel filtration chromatography. Whole-cell lysates were obtained from HeLa cells transfected with *Myc*-tagged *Smyd1* and *V5*-tagged *ASH2L* plasmids. The components of protein complexes were separated according to the differences in molecular sizes. Gel filtration assays showed that Myc-tagged Smyd1 and V5-tagged ASH2L eluted from the column in a close group (Figures 4B,C). The coexistence of Smyd1 and ASH2L was confirmed by Western blot using anti-Myc and anti-V5 antibodies to examine the eluted complexes. Co-immunoprecipitation assays were performed to further confirm that result. It turned out that V5-tagged ASH2L co-precipitated Smyd1 with the anti-V5 antibody but was not co-precipitated with the control IgG (Figure 4D). In addition, immunofluorescence microscopy revealed co-localization of endogenous Smyd1 and ASH2L in mouse heart (Figure 4E).

To define if the interaction between ASH2L and Smyd1 will promote the function of Smyd1, the *ASH2L* expression plasmid was cotransfected with the *Smyd1* and *pLG3-Isl1-WT* reporter into C2C12 cells. We found that in a suboptimal dosage, ASH2L slightly activated the *Isl1* promoter, while overexpression of ASH2L with Smyd1 dramatically increased *Isl1* promoter activity to 25-fold above basal level. Furthermore, the result of ASH2L cotransfected with HMTase-inactive mutant Smyd1 (Y234F) and the pLG3-Isl1-WT reporter showed that the mutant Smyd1 failed to induce Isl1 expression (Figure 4F). Taken together, these findings suggest that both the HMTase-inactive domain and ASH2L are required for fully activating the *Isl1* promoter.

### Smyd1 Bound to the *ANF* Promoter

**FIGURE 5:**
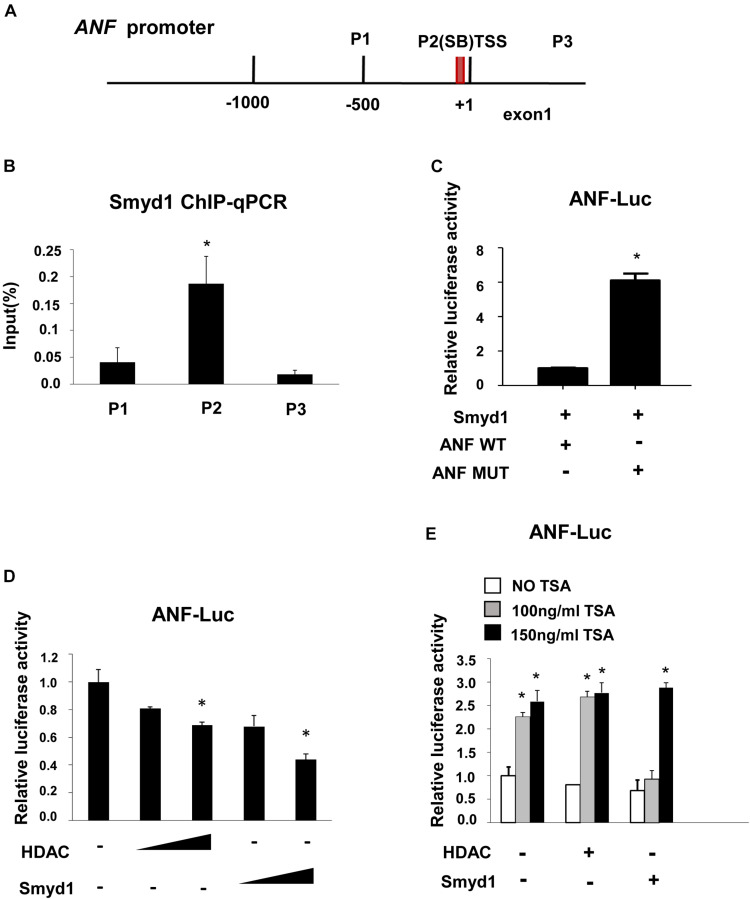
Smyd1 associates with HDAC to repress ANF expression. **(A)** The locations of the primer sets used for Smyd1 ChIP-qPCR are indicated. **(B)** The ChIP-qPCR results revealed that Smyd1 bound to the P2 region avidly. **(C)** Smyd1 expression vectors were cotransfected with pLG3-ANF-WT or mutant reporter (ANF MUT) into CV1 cells. **(D)** pLG3-ANF vectors were cotransfected with a different dosage of HDAC1 or Smyd1 into CV1 cells. **(E)** A different dose of TSA was added to pLG3-ANF/HDAC1 or pLG3-ANF/Smyd1-cotransfected cells. Data are shown as mean ± SD for three independent experiments. *p < 0.05 (Student’s *t* test).

*In situ* hybridization and qPCR results showed that *ANF* was upregulated in Smyd1 ablated heart (Rasmussen et al., 2015). To define if ANF expression was directly regulated by Smyd1, the ChIP-qPCR approach was carried out to examine the association between the Smyd1 and *ANF* promoter. As mentioned before, 5′-CCCTCC-3′ and 5′-GGAGGG-3′ are the candidate-binding elements for Smyd1. Looking through 1.0 kb upstream to TSS of the *ANF* promoter, we defined one conserved SB (5′-GGAGGG-3′) site located at −74 to −69 bp upstream to TSS. Primers were designed to cover that region (Figure 5A). ChIP analysis revealed that Smyd1 selectively bound to the SB2 site, but not the other regions (Figure 5B). We also generated substitution mutation of *ANF* on SB2 and found that mutation of the SB2 sequence abolished Smyd1-mediated repression of the pGL3-*ANF* activity (Figure 5C). Thus, SB2 is important for Smyd1-dependent transcriptional activity of the *ANF* gene, which is consistent with the ChIP results.

### Smyd1 Was Associated With HDACs to Repress *ANF* Expression

Since *ANF* was upregulated with Smyd1 deletion (Rasmussen et al., 2015), we proposed that *ANF* should be repressed in the presence of Smyd1. To test this possibility, a *pGL3-ANF* luciferase reporter which contains −700 to 0 region of the *ANF* promoter was cotransfected with *Myc-Smyd1* expression plasmid into CV1 cells. Smyd1 significantly reduced *ANF* promoter activity in a dose-dependent manner (Figure 5D). Smyd1 was shown to repress gene transcription in the presence of HDAC in yeast two-hybrid systems (Gottlieb et al., 2002). However, there was no report about Smyd1 target genes. *ANF* might be a candidate for Smyd1-dependent silencing. We asked whether HDAC members worked with Smyd1 on the *ANF* promoter. *HDAC1, 5, 9*, and *10* were transfected with the *pLG3-ANF* vector to CV1 cells separately. All of the HDACs inhibited *ANF* activity in a dose-dependent manner (data were not shown). To observe the potential synergy between Smyd1 and HDAC, we wanted to find out an optimal dosage of Smyd1. However, even a tiny amount of Smyd1 plasmid (20 ng per well of 12 wells plate) induced a dramatic decrease in ANF (50%). This is not surprising because CV1 cells expressed endogenous HDACs that may already be saturated. Then, we had to test it in another way. TSA, the HDAC inhibitor, was added to the cotransfection system at a concentration of 100 ng/ml. The inhibition from Smyd1 decreased to 50% compared to the non-TSA control. When the concentration of TSA was increased to 150 ng/ml, the inhibition caused by Smyd1 was completely abolished (Figure 5E). So, the repressor function of Smyd1 on the *ANF* promoter was at least partially due to the association with HDACs.

## Discussion

### *Smyd1* Regulated *Isl1* Activity

Isl1, a pivotal factor in the development of the SHF, also marks pluripotent cardiac progenitors with flk1 and Nkx2.5, which could be isolated, propagated, and differentiated into endothelial cells, smooth muscle cells, and cardiomyocytes (Kattman et al., 2006; Yi et al., 2017). Recent study showed that Isl1 can produce notable therapeutic effects in the infarcted heart (Li et al., 2017) and that Isl1-cardiovascular progenitor cells repaired after myocardial infarction (Bartulos et al., 2016). Isl1 is critical in early heart morphogenesis, but the upstream initiation mechanism of *Isl1* is not clear. Elucidation of the upstream factors of *Isl1* would help us to understand the mechanisms that initiate cardiac progenitor proliferation, survival, and migration, therefore providing us new strategies in treatment of cardiac disease.

The present study demonstrated that Smyd1 directly targeted and activated *Isl1* expression. In *Smyd1-Cko*-mutant mice, *Isl1* was dramatically downregulated in pharyngeal endoderm and splanchnic mesoderm, where the second heart progenitor cells derived from Cai et al. (2003). *Isl1* is required for cardiac progenitor proliferation. Consistent with that, our previous research showed that *Smyd1-Cko* mutant embryos exhibited decreased cell proliferation in pharyngeal endoderm and splanchnic mesoderm (Rasmussen et al., 2015). Therefore, the truncated outflow tract and right ventricle might be partly due to the decreased *Isl1* expression.

Global mapping of H3K4me3 sites shows that they are highly enriched in the proximal promoter of the expressed genes (within 1 kb of known or predicted transcript start sites) (Bernstein et al., 2006; Guenther et al., 2007). Moreover, H3K4me3 sites often overlap with CpG islands (Wei et al., 2009). However, the situations with specific events still remain elusive. Our Smyd1 ChIP-qPCR analysis showed that the binding between Smyd1 and the *Isl1* promoter was richest in the P4 region of the *Isl1* promoter (around −1200 to −1000 upstream to the TSS), which is much stronger than the bindings on TSS and other regions. Furthermore, the H3K4me3 ChIP-qPCR analysis revealed that the H3K4me3 level in the P4 region was significantly reduced with no big effect on the TSS region. Those data suggested that Smyd1 was responsible for the H3K4me3 of the P4 site, but not that of the TSS. One question about those results is why no change of H3K4me3 was observed in the TSS region. It is possible that some other methyltransferases work on the TSS region of the *Isl1* promoter. This may also explain why the expression of Isl1 was partially downregulated but not totally turned off in *Smyd1-Cko* mutant embryos.

The research on Smyd3, a family member of Smyd1, reveals that Smyd3 could specifically bind to 5′-CCCTCC-3′ or 5′-GGAGGG-3′ motifs on the promoter region of *Nkx2.8* and *hTER* genes to direct oncogenesis (Hamamoto et al., 2004; Liu et al., 2007). Although the expression pattern of *Smyd1* and *Smyd3* is different, they are highly homologous in structure, suggesting that Smyd1 has the same DNA-binding potential as Smyd3. Our EMSA assays demonstrated that Smyd1 bound to the SB4 site of the P4 region avidly. In addition, Smyd1 could activate *pLG3-Isl1* Luc promoter activity alone. Site mutation on the SB4 site in the P4 region blocked the *pLG3-Isl1* Luc reporter activity. Those data strongly support the premise that Smyd1 not only has the methyltransferase potential but also has the ability to bind to a specific binding motif on the promoters of target genes. This may be a common character of Smyd family members, which is different from another methyltransferase, such as MLL1. Based on the specific DNA-binding potential of Smyd1, one possible mechanism is the high-level H3K4me3 at the P4 region which facilitated the reorganization of the *Isl1-*targeted motif by another transcriptional factor. A reference example is transcription factor Myc (Guccione et al., 2006). Research shows that the recognition of Myc-binding sites is based on epigenetic features. A chromatin bearing high H3K4/K79 methylation and H3 acetylation was essential for Myc to bind DNA. It suggests that chromatin recognition is ahead of the specific binding sequence recognition. Based on that, one hypothesis is that the H3K4me3 induced by Smyd1 may open the chromatin and recruit transcriptional factors to bind to adjacent motifs on the *Isl1* promoter. Since the regulatory mechanisms underlying the *Isl1* expression are largely unknown, we have few confirmed information about the transcriptional factors which regulate *Isl1*. In addition, our data demonstrated that Smyd1 interacted with ASH2L. Hence, it is possible that ASH2L may bridge Smyd1 to work with other factors to regulate gene activation. So far, the association between Smyd1 and H3K4me3 on the *Isl1* promoter may function as a key event for cascade initiation of *Isl1* promoter.

### Smyd1 Directly Repressed the Expression of ANF

Developmental processes are always dynamic (Bondue et al., 2008), during which Smyd1 negatively regulated the expression of approximately hundreds of genes (Rasmussen et al., 2015). Among them, we specifically examined *ANF*. In *Smyd1-Cko* embryos, *ANF* was upregulated in the left ventricle and atrium (Rasmussen et al., 2015). The Smyd1-ChIP-qPCR analysis revealed the binding of Smyd1 to the *ANF* promoter. Furthermore, the inhibition of ANF by Smyd1 was at least partially through the association with HDACs. Those data strongly suggest that Smyd1 directly controls the transcription of *ANF*. In addition, *BNP* and *β-MHC* were all upregulated in *Smyd1-Cko* embryos. *β-MHC* was also enhanced in the left ventricle and atrium region (Rasmussen et al., 2015). The potential connection of those changes might be the expansion of cell populations other than the SHF progenitor cells. Repression of those genes by Smyd1 may ensure that the balance between different cell populations is precisely regulated by Smyd1 at the developmental stage.

### Future Considerations

Our current study identified Smyd1 as a critical epigenetic factor in early heart morphogenesis through both positive and negative regulation. Smyd1 decreased the expression of key factors, which directs cardiac progenitor cell development, such as Isl1. To examine the exact function of Smyd1 in directing the cardiac progenitor cell population, the overexpression experiments in ES cells are being undertaken. Meanwhile, evidence showed that Smyd1 carried out diverse functions on different gene promoters through association with cofactors, including ASH2L, HSP90α, HDAC, and skNAC. In the future work, it is worthy to find out more cofactors of Smyd1 and how they cooperate with Smyd1 to control gene expression.

In summary, our studies provided new insights about the functional mechanisms of Smyd1 in heart development. Smyd1 activated the expression of *Isl1* and inhibited *ANF* transcription. The interaction between Smyd1 and other cofactors may help it to perform those different functions under various conditions, such as ASH2L and HDAC. More factors involved in this machinery are waiting to be elucidated.

## Data Availability Statement

The original contributions presented in the study are included in the article/supplementary material, further inquiries can be directed to the corresponding authors.

## Ethics Statement

The animal study was reviewed and approved by the Ethical Committee of The First Affiliated Hospital of Hainan Medical University.

## Author Contributions

RS and YM designed the research. YM, ZW, QL, and JL performed the research work. YM, ZW, and FS analyzed the results. YM, ZW, and QL wrote the manuscript. All authors contributed to the article and approved the submitted version.

## Conflict of Interest

The authors declare that the research was conducted in the absence of any commercial or financial relationships that could be construed as a potential conflict of interest.
